# Progress and Challenges in Elucidating the Functional Role of Effectors in the Soybean-*Phytophthora sojae* Interaction

**DOI:** 10.3390/jof9010012

**Published:** 2022-12-21

**Authors:** Mst Hur Madina, Parthasarathy Santhanam, Yanick Asselin, Rajdeep Jaswal, Richard R. Bélanger

**Affiliations:** Département de Phytologie, Université Laval, Québec, QC G1V 0A6, Canada

**Keywords:** hemibiotrophs, effector, localization, protein–protein interactions, structures, soybean

## Abstract

*Phytophthora sojae*, the agent responsible for stem and root rot, is one of the most damaging plant pathogens of soybean. To establish a compatible-interaction, *P. sojae* secretes a wide array of effector proteins into the host cell. These effectors have been shown to act either in the apoplastic area or the cytoplasm of the cell to manipulate the host cellular processes in favor of the development of the pathogen. Deciphering effector-plant interactions is important for understanding the role of *P. sojae* effectors in disease progression and developing approaches to prevent infection. Here, we review the subcellular localization, the host proteins, and the processes associated with *P. sojae* effectors. We also discuss the emerging topic of effectors in the context of effector-resistance genes interaction, as well as model systems and recent developments in resources and techniques that may provide a better understanding of the soybean-*P. sojae* interaction.

## 1. Introduction

*Phytophthora sojae* Kaufm. & Gerd., a hemibiotrophic oomycete, is one of the most destructive pathogens of soybean [1]. Along with other oomycetes, it is a member of the Stramenopila kingdom, which is evolutionarily distant from the kingdom Fungi [2,3]. Being a hemibiotroph, *P. sojae* initially establishes an intimate association with living host tissues and later, enters a necrotrophic phase where its colonization leads to cell death [4]. 

**Figure 1:**
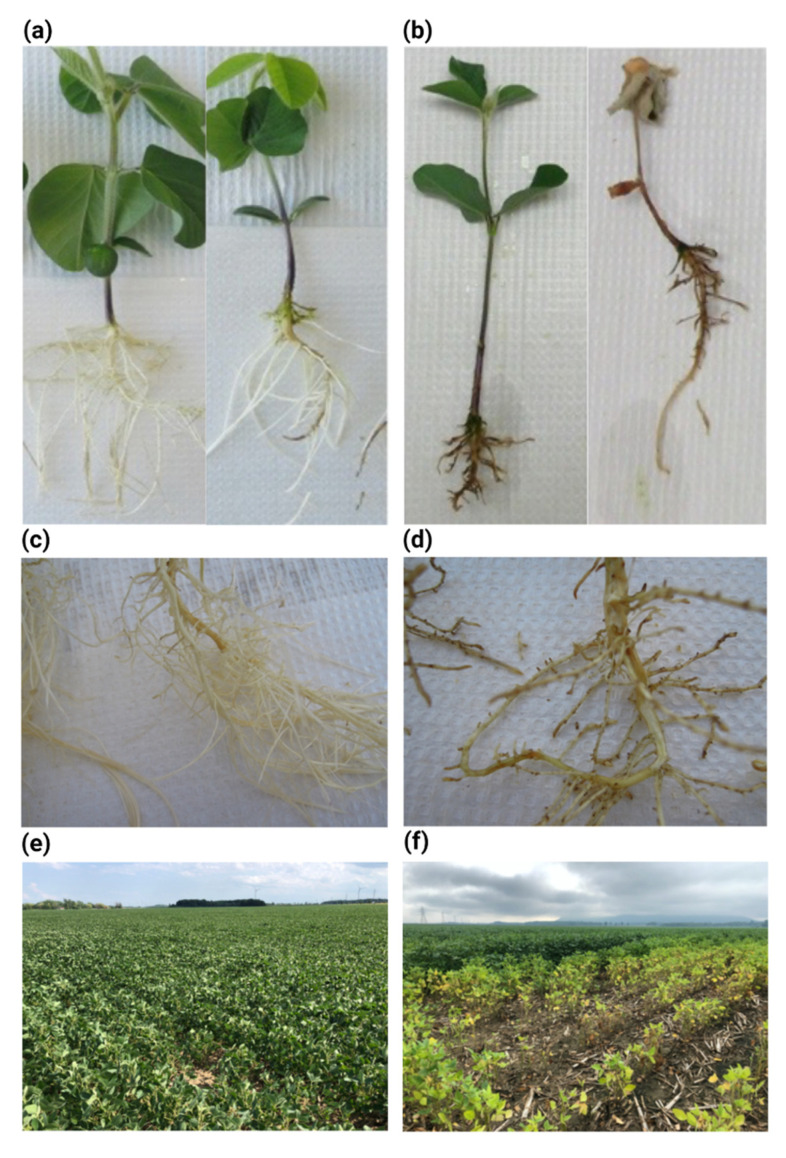
Disease symptoms of *Phytophthora sojae* on soybean. Illustrations of (**a**) control seedlings and (**b**) infected seedlings and (**c**) a closer look at healthy roots and (**d**) rotten roots. Typical soybean field showing (**e**) healthy and (**f**) infected plants.

*Phytophthora sojae* can infect soybean plants throughout the growing season from the time of planting until harvest. The infection process starts in the roots and ultimately spreads to invade the plant stem ([4]; Figure 1). It can cause seed rot before emergence, plant wilt after emergence, and leaf wilting and yellowing during the adult plant stage ([5]; Figure 1). The yield losses might reach 100% if susceptible cultivars are planted in fields with poor drainage that have a history of the disease. The economic damages associated with *P. sojae* are estimated approximately to 1.2 million Mt in the USA and around $1–2 billion worldwide [5,6]. Despite the importance of *P. sojae*, the molecular details of its pathogenesis and interaction with resistance genes (*Rps*) remain elusive. As one of the first sequenced oomycete pathogens [7], the study of *P. sojae* has been facilitated by the abundance of genetic and genomic resources. For instance, the recently developed CRISPR/Cas9-mediated genome editing technology in *P. sojae* allows high-throughput functional genomic research [8].

In this review, we describe the current state of understanding regarding the soybean-*P. sojae* interaction with a focus on the subcellular localization, the targeted host proteins and potential cellular processes exerted by *P. sojae* effectors. We then illustrate how the CRISPR, and hairy root techniques offer a tremendous lever to foster a better appreciation of the function of *P. sojae* effectors. We also exploit the network AlphaFold to offer predictive and comparative insights into the structures of *P. sojae* effectors. Finally, we briefly discuss the effector-resistance gene interactions and address the next challenges to overcome in the study of *P. sojae* effectors. 

## 2. Effector Proteins of *P. sojae*: The Key to Successful Infection

During the arms race between a pathogen and its host, the host produces defense responses to fend off the pathogen while the pathogen also utilizes a wide array of strategies to infect the plant, one of which being the secretion of effector proteins. Although the term “effector” is widely used in the plant-microbe interaction research field, its definition varies. Pritchard et al. (2014) defined effectors as “any protein synthesized by a pathogen that is transported into the host and has an impact of making the host environment favorable to the pathogen” [9]. However, the widely accepted hallmark of effectors includes small-secreted proteins, having an N-terminal signal peptide with low sequence similarity across species and the ability to modify host cellular and molecular processes to facilitate infection as virulence factor or triggering defense responses as avirulence (Avr) factor [10]. Like other filamentous pathogens, *P. sojae* uses a specialized structure called haustorium that invaginates the plasma membrane of soybean root cells and secretes effector proteins to manipulate plant immunity and establish a sustainable biotrophic colonization. The genomic data suggests that *P. sojae* produces many secreted effector proteins that may be classified as apoplastic or cytoplasmic based on their putative localization in a plant cell [1,11,12].

Apoplastic effectors are secreted into the apoplast of plant cells, and they bind either host derived apoplastic proteins or membrane-localized pattern recognition receptors (PRRs), acting either as virulence factors to inhibit plant defenses or as molecular patterns to stimulate plant immunity [13]. The majority of *P. sojae* apoplastic effectors reported to induce host cell death belong to the glycoside hydrolase (GH) and Nep1-like proteins (NLPs) protein family [14]. The NLPs contain an N-terminal secretion signal and a semi-conserved necrosis-inducing *Phytophthora* protein 1 (NPP1) domain [15]. The genome of *P. sojae* contains 70 putative NLP genes, 33 of which are anticipated to encode for authentic NLPs [16].

Cytoplasmic effectors are translocated into the plant cytoplasm from where they are directed to various cellular compartments and modulate a broad spectrum of cellular processes such as plant immunity, physiology, and metabolism in favor of microbial growth [17,18,19]. The *P. sojae* genome encodes ∼600 cytoplasmic effectors [7,11] divided into two types based on the sequence motifs, namely RxLRs and CRNs (Crinkling and Necrosis Inducing Proteins). The RxLR effectors contain an N-terminal signal peptide for protein secretion, a conserved N-terminal RxLR motif to facilitate translation into host cells, and a variable C-terminal domain responsible for virulence activity [7,11]. The RxLR effector family has been reported as the largest class of translocated effectors in *Phytophthora* spp., and *P. sojae* contains~390 of them [7,20,21]. The CRNs belong to another important effector family uniquely produced by oomycetes [22,23,24]. They were first identified in the plant pathogenic *Phytophthora infestans* [25]. Like RxLR proteins, CRNs have a highly conserved N-terminal containg LxLFLAK, HVLVXXP, and DWL domains, which facilitate translocation of the CRN proteins from the apoplast into the plant cytoplasm [11]. This is followed by a variable C-terminal that conveys different functions, including subcellular localization required for the effector function. In *P. sojae,* the average expression levels of CRNs are substantially higher than those of RxLRs during the infection, suggesting a crucial virulence function [26].

As with effectors from other pathogens, *P. sojae* effectors are translocated toward many subcellular organelles and structures, where they target a wide range of host proteins and pathways within the plant cells [27,28]. In recent years, there has been significant progress in the characterization of *P. sojae* effectors to understand the importance of these proteins in pathogenesis.

## 3. Where to Go: *P. sojae* Effectors Target Multiple Plant Cell Compartments

**Figure 2:**
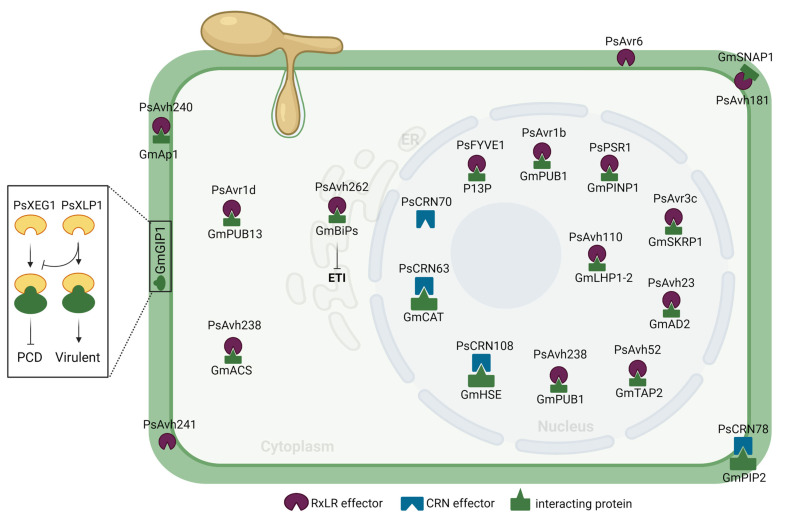
Subcellular localization and corresponding host targets of *Phytophthora sojae* candidate secreted effector proteins (CSEPs) based on current literature. The insert to the left depicts the effector PsXEG1 and its decoy PsXLP1 binding to GmGIP1 in the apoplast to create an incompatible (programmed cell death (PCD) or a compatible (Virulent) interaction, respectively.

Several studies have shown that the distribution of effectors in a specific cell compartment is critical for their action in plants during infection [29,30,31]. To date, only a handful of *P. sojae* effectors (∼20) have been the subject of localization experiments. Interestingly, the conclusions showed that each one targeted a different plant cell compartment (Table 1; Figure 2). Most of the studies were performed in heterologous plant systems (*A. thaliana* or *N. benthamiana* or onion epidermal cells) where effector proteins were expressed without their signal peptide and fused to the green fluorescent protein (GFP). However, immunolocalization was recently used to study *P. sojae* effector subcellular localization, one of the rare instances succeeding in labeling effectors in situ [32]. Based on the GFP fusion system, the majority of *P. sojae* effectors are found to be localized in the nucleus of plant cells (Figure 2) and this applies in particular to CRNs, which contain a conserved nuclear localization signal or sequence (NLS) [33,34,35,36,37,38,39,40,41,42,43,44]. As such, it is believed that these effectors are required to localize into the nucleus to be functional [37,38,39,45,46]. However, a recent study has shown that one of the CRN effectors, PsCRN78, executes its molecular activity along the plasma membrane of the plant cell, in contradiction to the specified nucleus localization function [47].

On the other hand, several RxLR effectors of *P. sojae* have been found to be localized in the plasma membrane (Figure 2) through N-myristoylation modification, a novel signal for membrane targeting [51,52,55,56]. Despite the absence of a transmembrane domain and a predicted myristoylation modification site, PsAvh240, a *P. sojae* effector, localized to the plasma membrane and retained its pathogenicity function [50]. It is probable that the positively charged patch interface of PsAvh240 may interact with the negatively charged electrostatic field on the surface of the plasma membrane inside the cell, thus resulting in the membrane localization of PsAvh240. Alternatively, PsAvh240 may localize to the plasma membrane through an interaction with a membrane protein via its two-helix motif. Further research is necessary to elucidate the process by which the two-helix motif would promote this specific localization. However, some effectors have also been found to be localized in the apoplast or intracellular space [32,48,49]. A recent immunolocalization study has revealed that the PsAvr6 effector was localized exclusively in the apoplastic area [32].

*Phytophthora sojae* effectors are not only directed to the plasma membrane, apoplast, cytoplasm, or nucleus, but also to the endoplasmic reticulum (Figure 2) [53]. PsAvh262, for example, targets the ER, a critical location for the modification and fold of secretory and membrane proteins in eukaryotic cells [57,58].

### Fusion Tagged Localization in Heterologous Systems, Right Choice or Not?

As mentioned earlier, most studies of cellular localization of *P. sojae* effectors were performed with heterologous expression systems, including *A. thaliana*, *N. benthamiana* or onion epidermal cells. Heterologous expressions are often used to take advantage of analytical tools that are not available in the organism where the gene and its product originate or have an impact. Although stable or transient expression in model systems is faster, and easier, it might lack the important components of the native systems, such as incorrect protein folding leading to mis-localization within the cell.

To access localization, effectors have been expressed without signal peptide *in planta*. Although the signal peptide is believed to be cleaved upon secretion and not present in the mature protein [23,55,59], effectors should be expressed with or without signal peptide to reduce biases towards the localization or other experimental outcomes. Moreover, several *P. sojae* effector proteins have been shown to localize to the nucleus based on N-terminal GFP-tagged assays [33,34,37,38,39,40,44]. It should be noted that GFP fusion may impair proper effector localization, either by hiding a sorting signal or by altering the three-dimensional structure of native effectors, preventing the interaction with a protein required in true effector localization. Therefore, although GFP-tagging is a highly effective tool for identifying an effector’s subcellular localization, caution should be taken when interpreting the results. Furthermore, many proteins carry localization signals at their N-terminal region. A fusion tag attached to the N-terminal of a protein may prevent the function of the signal sequence and cause mis-localization. Indeed, some evidence exists that placing a frame fusion tag at the C-terminal fusion of a protein is more effective than placing tags at the N-terminal [60]. However, some studies have suggested that the C-terminal of RxLR proteins is important for their effector functions [28,61]. Therefore, to avoid interfering with the N- or C-terminal effector domain, it would be better to add both N- and C-terminal fusion tags with the interested protein and test them for localization independently.

For a long time, protein localization has been assayed mainly by conventional methods including biochemical fractionation and microscopic imaging. However, recent reports highlight significant progress in the use of affinity purification coupled with mass spectrometry (AP-MS) in studying subcellular protein localization and protein–protein interactions (PPIs) [62,63,64,65]. The AP-MS is a fast and sensitive method and can be applied to large-scale studies and has been demonstrated to have high intra-and inter-laboratory reproducibility. Since the AP-MS purification is performed under non-physiological conditions in lysed samples, it is inevitable to lose weak and transient associations or to give a false-positive result [66,67]. Recently, to overcome some of these drawbacks, enzyme-catalyzed proximity labeling (PL) approaches such as BioID, TurboID, APEX have been developed to study the interactions and localization of endogenous proteins in under native conditions in living cells. Through proximity labeling, an engineered enzyme is targeted to a specific protein or subcellular compartment by genetic fusion. Neighboring proteins are labeled by a promiscuous enzyme, that allows protein purification and identification. The second-generation enzymes APEX2 and TurboID, have been recently developed with enhanced catalytic efficiency allowing either increase labelling activity or decrease labelling time [68,69]. Both AP-MS and PL-MS are powerful techniques that significantly enhance our understanding simultaneously both protein localization and expression in plant cell, could be used for characterizing *P. sojae* effectors. Additionally, every method has its own pros and cons. In the future, it’s likely that new enzymes will be developed and that existing systems like BioID, TurboID, and APEX will be improved to make them more useful to address a range of biological questions including the constituency of the subcellular compartment, protein structures, localizations, and interactions between proteins.

## 4. Which Proteins and Processes Do *P. sojae* Effectors Target?

*Phytophthora sojae* effectors, like those from other fungi and oomycetes, target a variety of host proteins affecting stability inside the cell [70]. This association may alter a diverse range of cellular processes including plant cellular metabolic pathways, signalling and trafficking cascades, and interference with DNA machinery [71].

### 4.1. Modulation of Apoplastic Immunity

The most studied apoplastic effector of *P. sojae* is PsXEG1 (Xyloglucan-specific endoglucanase 1), a member of the GH12 (glycoside hydrolase 12) family [48,49]. Both in vivo and in vitro assays have shown that PsXEG1 interacts with the soybean apoplastic glucanase inhibitor protein (GmGIP) (Figure 2). The soybean GmGIP1 strongly suppresses PsXEG1 hydrolase activity and blocks its involvement in virulence [49]. However, *P. sojae* releases a paralogous PsXEG1-Like Protein (PsXLP1) that lacks enzymatic activity but has a substantially higher affinity for the GmGIP1 than does PsXEG1. Therefore, PsXLP1 effectively competes with PsXEG1 to bind with GmGIP1 hence freeing PsXEG1 to support *P. sojae* infection. Notably, PsXLP1 overexpression in soybean promotes *P. sojae* infection only when PsXEG1 is functional, implying that PsXLP1 functions as a ‘pseudo-effector,’ releasing PsXEG1 from GmGIP1 suppression rather than as a true virulence effector. Both PsXLP1 and PsXEG1 are in close proximity to one another in the genome and share the same promoter.

Another virulence effector, PsAvh240, has been shown to interact with soybean aspartic protease, GmAP1, and blocks its secretion into the apoplast, hence supressing the apoplastic defense response and promoting the infection of *P. sojae* (Figure 2) [50]. The plasma-membrane localized effector, PsAvh181, was recently found to interact with a soluble N-ethylmaleimide-sensitive factor (NSF) attachment protein, GmSNAP1 in soybean [52]. Notably, PsAvh181 interferes with the interaction between GmSNAP1 and GmNSF and blocks the secretion of apoplastic defense-related proteins, such as pathogenesis-related protein PR-1 and apoplastic proteases [52].

### 4.2. Regulation of Programmed Cell Death in Plant

Like other hemibiotrophic pathogens, *P. sojae* initially feeds on living host tissues and later becomes necrotrophic by killing plant cells. Thus, the fine control of plant programmed cell death is essential to establish a successful infection.

PsAvh262, a *P. sojae* RxLR effector, targets and associates with the plant endoplasmic reticulum (ER)-luminal binding proteins (BiPs), which serve as negative regulators of plant immunity. PsAvh262 stabilizes GmBiP1 and suppresses ER stress-triggered cell death (Figure 2) and facilitates *P. sojae* infection [53]. Another RxLR effector, PsAvh238, binds to 1-aminocyclopropane-1-carboxylic acid synthases (ACSs) and inhibits their activity via a 26S proteasome-dependent destabilization of the protein and triggers host cell death [44,71].

Unlike the above mentioned RxLR effectors, PsCRN63 and PsCRN115 are two homologous CRN effectors that have opposing functions in controlling PCD; however, these effectors are needed jointly for virulence of *P. sojae* (Figure 2) [39]. Both effector proteins interact directly with plant catalases (CATs) and alter hydrogen peroxide (H_2_O_2_) homeostasis, hence modulating host cell death [39]. Another CRN effector, PsCRN78, was reported to interact with aquaporin PIP2-family proteins including NbPIP2;2 from *N. benthamiana* and GmPIP2-13 from soybean (Figure 2) [47]. Using its kinase domain, PsCRN78 promotes the phosphorylation of NbPIP2;2 or GmPIP2-13 in vivo, resulting in their subsequent degradation through a 26S-dependent pathway. NbPIP2;2 functions as a H_2_O_2_ transporter, enhancing plant immunity and reactive oxygen species accumulation (ROS).

Recently, PsAvr1b was reported to inhibit host cell death by interacting with the U-box soybean protein GmPUB1-1 (Figure 2) [42]. The report showed that GmPUB1-1 might be a key regulator of effector-triggered immunity (ETI) that is shielded against PsAvr1b-binding by proteins encoded by genes within the *Rps1-b* and *Rps1-k* loci. Later, it was found that GmPUB1-1 interacts with a variety of other RxLR effectors, including PsAvr1d, whereas PsAvr1b and PsAvr1d interacted with a variety of other infection-induced GmPUB proteins, indicating that the pathogen modulates host immunity via a multiplex interaction of RxLR effectors with GmPUB proteins [42,54].

### 4.3. Modulation of Gene Regulation System in Plants

Plants must have strict regulation over the defense-related gene expression to mount effective defenses (Jenner and Young, 2005). Reprogramming host gene expression appears to be a common virulence strategy used by microbial pathogens [33,34,35,36,38,72].

PsCRN108 is a CRN effector required for virulence that has a putative DNA-binding helix-hairpin-helix (HhH) motif [38]. PsCRN108 binds directly to heat shock elements (HSEs) in the promoter region of several plant heat shock protein (HSP) genes (Figure 2), depending on their nuclear localization and the HhH motif [38]. Heat shock proteins are a class of molecular chaperones that play a significant role in basal defenses in the plant [38,73,74]. It is well known that the expression of HSP genes in plants is controlled by upstream heat shock factors (HSFs) via binding to heat shock elements (HSEs) in the HSP promoter [75]. Since PsCRN108 binds to HSEs (Figure 2), which interrupts the binding of endogenous transcription factors to HSEs, this thereby blocks the transcriptional activation of HSP genes involved in defense responses against *Phytophthora* spp. The PsAvr3c effector interacts with and stabilizes the soybean serine/lysine/arginine-rich proteins (GmSKRPs), which are components of the plant spliceosome and regulate host pre-mRNA splicing in order to modulate plant immunity [33]. Both PsAvr3c and GmSKRPs are known to regulate a broad spectrum of host gene alternative splicing (AS) events (Figure 2), demonstrating that PsAvr3c uses GmSKRPs as a virulence target to change the plant AS machinery. Another *P. sojae* effector, PsFYVE1, targets spliceosome components of hosts soybean (GmRZ-1A/1B/1C) and *N. benthamiana* (NbRZ-1A/1B/1C) and regulate plant defense related genes at both the transcriptional and AS levels during the interaction with soybean [43]. PsPSR1 and PsPSR2, two unrelated RxLR effectors, have been found to control RNA silencing in plants by blocking sRNA synthesis [35]. Overexpression of PsPSR effector in *N. benthamiana* significantly increased *Phytophthora capsici* infection, indicating that disrupting the plant RNA silencing machinery is a critical counter-defense strategy of *P. capsici*. The PSR1 is a general suppressor of sRNA accumulation and interacts directly with PSR1-interacting protein 1 (PINP1), a nuclear protein containing the aspartate-glutamate-alanine-histidine (DEAH)-box RNA helicase domain in *Arabidopsis* (Figure 2) [36]. PINP1 controls both miRNA and endogenous siRNA accumulation and serves as a positive regulator of plant immunity. By silencing PINP1, it was possible to disrupt the formation of microRNA-processing complexes in the nucleus, resulting in developmental defects and poor plant immunity. This demonstrates that PINP1 is a direct target of PsPSR1 in mediating plant sRNA accumulation and immunity, even though the mechanisms by which PsPSR1 controls PINP1 to trigger plant immunity suppression is unclear. It was recently shown that a *P. sojae* RxLR effector PsAvh23 disrupts histone acetylation modification process to promote infection [34]. The PsAvh23 interacts with the ADA2 subunit of the histone acetyltransferase (HAT) complex Spt-ADA-Gcn5-acetyltransferase (SAGA) in plants (Figure 2). ADA2 is an essential regulatory subunit of the GCN5 HAT complex, and forms a subcomplex with GCN5, which interacts with transcriptional factors to promote gene activation [76]. Interestingly, PsAvh23 has the same binding sites on GmADA2-1 as does GmGCN5, and by competing for the GmADA2 binding site, PsAvh23 mitigates the ADA2-GCN5 interaction [34]. This is supported by the fact that Avh23 inhibits GmADA2-1-mediated HAT activity and decreases the acetylation levels of H3K9. PsAvh23 overexpression or silencing of GmGCN5 also results in the suppression of a large number of defense-related genes. Together these data indicate that Avh23 down-regulates plant defense-related gene expression by hijacking ADA2 to reduce GCN5 HAT activity, probably by destroying the ADA2-GCN5 subcomplex. Another *P. sojae* effector, PsAvh52, interacts with GmTAP1, a new acetyltransferase, enabling GmTAP1 to move from the cytoplasm to the nucleus. GmTAP1 acetylates core histones in the nucleus, upregulating the expression of possible plant susceptibility genes and promoting bacterial pathogenic infection successfully [41].

## 5. The Structure of *P. sojae* Effectors

Exploring the structure of effector proteins is critical to understand the molecular mechanisms behind the pathogenic and symbiotic interaction processes. For this purpose, genome-wide characterization of effector protein genes and proteins provides a great insight into their functional roles, conserved sequence motifs, and evolutionary patterns [77]. Moreover, predicting and identifying how an effector interacts with host target proteins opens opportunities to understand the related pathways involved, and ways of manipulating them to establish plant protection [71,78].

So far, PsAvh240, PsPSR2, and PsAvr1d are the only three effector structures in *P. sojae* that have been resolved experimentally using X-ray crystallography methods [50,54,79]. The crystal structure of PsAvh240 revealed that it contains six α helices and two WY motifs. Its first two α helices are necessary for its plasma membrane localization and interaction with GmAP1. The second WY motifs of two PsAvh240 molecules create a handshake configuration, resulting in a dimer conformation. The structure of the PsPSR2 effector was also shown to contain a WY motif including six variant WY motifs (termed LWY motif), and this arrangement was detected in 293 RxLR effectors from five *Phytophthora* species [79]. A more recent study demonstrated that the crystal structures of PsAvr1d provide a structural basis showing how it interacts with its target GmPUB13, functioning as a susceptibility factor for plants. The structure of this effector has been shown to contain a single WY motif and three α-helix bundles in which the Tyr-118 and Trp-96 are able to form a hydrophobic core facilitating the interaction with GmPUB13. It has been demonstrated that Phe-90 is the predominant amino acid in this interaction, inhibiting the ubiquitin ligase function of GmPUB13 and helping the infection process [54].

In support of structural studies of effector proteins, recent breakthroughs in the development of tools such as AlphaFold and RoseTTaFold have revolutionized the analysis of effectors, as these can predict the structure of unannotated candidate effector proteins [80,81]. Several recent analyses on the secretome of different plant pathogens such as *Magnaporthe oryzae*, *Fusarium oxysporum*, *Puccinia graminis*, and others have identified effector families possessing similar fold to known protein families having a role in plant-pathogen interactions or essential for pathogen virulence [82,83,84]. While these effectors have no sequence similarity, they bear a conserved three-dimensional fold of known effectors and have been designated as structurally similar sequence-unrelated effector proteins. The presence of conserved fold in these candidates can therefore be used to predict the function of the novel effector candidates [83].

**Figure 3:**
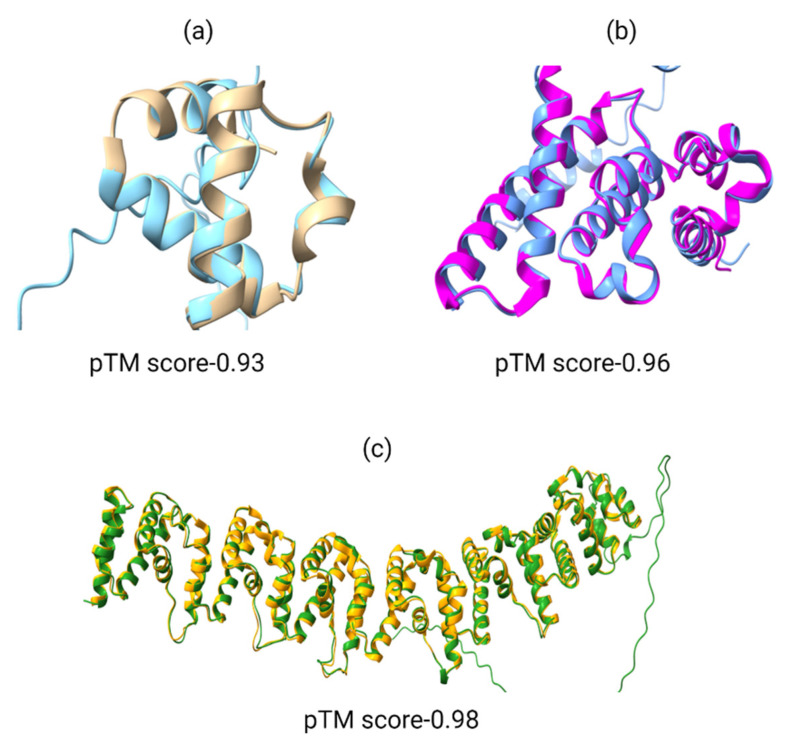
Comparison of crystal structures of avirulence proteins of *P. sojae* with AlphaFold predicted structures by superposition. As expected, the crystal and predicted structures were highly similar except at the disordered regions. (**a**) PsAvr1d comparison: golden color represents PsAvr1d crystal structure while cyan blue represents AlphaFold predicted structure of PsAvr1d. (**b**) PsAvh240 comparison: pink color represents the PsAvh240 crystal structure whereas blue represents the AlphaFold predicted PsAvh240 structure. (**c**) PsPSR2 comparison: yellow color represents the crystal structure whereas green represents the AlphaFold predicted structure of PsPSR2. pTM is template score on a scale from 0 to 1.

To our knowledge, no studies have yet exploited AlphaFold or RoseTTaFold for elucidating the structures of *P. sojae* effectors. Therefore, in this review we have tried the AlphaFold program for modelling of the structure of *P. sojae* effector proteins in order to assess the efficacy of AlphaFold to the effector field (Figure 3). We have chosen PsAvr1d, PsAvh240, and PsPSR2 effectors that already had 3D structures in the PDB database for practical reasons and compared the modelling results with the experimentally resolved protein structures. In our analysis, the AlphaFold network predicted the structures of three known effectors (PsAvr1d, PsAvh240, and PsPSR2) with a >90% accuracy including the prediction of disordered regions with a pTM score- >0.90 (Figure 3). The AlphaFold Model is the predicted TM-score (pTM) which reflects the overall topological accuracy of the predicted structure. The pTM score is calculated as predicted aligned error (PAE) and based on estimates of the error of the position of each amino acid in the final pair of the MSA generated (multiple sequence alignment). The pTM ranges from 0-1, where 0 represents minimal and 1 represents maximum accuracy.

## 6. Functional Characterization of Effectors in *P. sojae* Using CRISPR

Homologous recombination based targeted gene knockout has been widely used for functional characterization of different genes in a number of plant pathogens [85]. This approach was very successful in many model pathogens such as *Ustilago maydis* but did not work efficiently in oomycetes and other fungal pathogens with an active non homologous end joining (NHEJ) based DNA repair mechanism. Van West et al. (1999) successfully silenced *inf1* in *P. infestans* through internuclear gene silencing. Silencing *Avr4/6* using RNAi resulted in a gain of virulence of *P. sojae* isolates against *Rps4* or *Rps6* [86,87]. Qutob et al. (2013) silenced *Avr3a* in *P. sojae* by transgenerational gene silencing [88]. However, gene-silencing based on reverse genetics is still challenging in oomycetes and some fungal pathogens.

CRISPR-Cas9 technology has been successfully applied in various pathogens and plant species [89]. Using this approach, one can precisely manipulate a given target gene in the genome of interest. Since its initial report, CRISPR technology has been used to generate knockouts or knock-ins in more than 40 fungal and oomycete pathogens [90], including *P. sojae* [8] and *P. infestans* [91]. Once the Cas9 generates a double-strand DNA break in the target region, DNA repair mechanisms are activated [92,93] and the break repaired either by a dominant non-homologous end-joining mechanism (NHEJ) or homology-directed repair (HDR). DNA repair by NHEJ introduces insertions or deletions, which leads to frameshift mutations resulting in gene knock out. In some fungal species, where homology-directed repair is active, a double-strand DNA break will be repaired using a repair template (donor DNA) containing a selectable marker flanked by target regions.

As a result of CRISPR-based gene editing methodology, functional characterization of effectors is gradually increasing in *P. sojae*. Fang and Tyler (2016) overcame several technical hurdles to successfully implement CRISPR-Cas9 based genome editing in *P. sojae* [8]. They optimised NLS tag, identification of RNA pol III promoter and an additional self-cleaving ribozyme flanking the sgRNA. Following their success, several transformations using CRISPR-Cas9 based editing were demonstrated in *P. sojae* [34,54,94,95]. At the time of writing this review, all CRISPR-Cas9 based targeted gene knockouts or knock-ins in *P. sojae* were generated using the plasmids developed by Tyler’s lab [8].

*P. sojae* effector PsAvh23 suppresses histone acetylation resulting in misregulation of defense-related genes in soybean. Using CRISPR, *PsAvh23* gene was knocked out and replaced with NPTII, which resulted in upregulation of selected defense related genes [34]. Interestingly, when *PsAvh23* was overexpressed in the soybean hairy root system, defense-related genes were downregulated favoring pathogen virulence [34]. When overexpressed, effector Avr3c regulated soybean immunity by promoting alternate RNA splicing mechanisms by binding to GmSKRPs, a component of spliceosome [33]. In addition, knock out of *Avr1d* promoted virulence by suppressing the self-ubiquitination of soybean E3 ligase GmPUB13 [54]. Using the yeast two hybrid system, it was shown that Avr1b and Avr1d interacted with GmPUB1-1. Moreover, Avr1b and Avr1d interacted with several other biotic stress induced GmPUB proteins indicating modulation of host immunity by these effectors [42]. Knockout experiments using CRISPR showed that Avh181 was a virulence factor required for *P. sojae* infection [52]. The combination of Illumina based deep sequencing and long read sequencing by MinIon resulted in the identification of five copies of *Avr3a* in proximity. Functional analyses using CRISPR-Cas9 knockout and constitutive expression demonstrated that *Avr3a* interacted with *Rps8*. This new finding shows that *Avr3a* interacts with *Rps3a* and *Rps8* to trigger host immunity [94]. Even though chitin is not present in *P. sojae*, the pathogen does harbor two chitin synthase (CHS) genes (*PsCHS1* and *PsCHS2*) that are differentially regulated during the infection process. Knocking out *PsCHS1* and *PsCHS2* independently or simultaneously using CRISPR showed that only *PsCHS1*, is required for zoospore germination and hyphal branch formation during early infection [95]. It is interesting to see multiple RxLR effectors targeting single guard or helper proteins and single effector targeting multiple R proteins. Generation of double knockouts in *P. sojae* would not have been possible without CRIPSR-Cas9 technology. Targeting a family of genes or multiple unrelated genes is possible by multiplexing sgRNA. In *Fusarium fujikuroi*, three different genes were targeted simultaneously using the same Pol III promoter [96]. Similarly, in *U. maydis,* five members of *eff1* effector genes were knocked out simultaneously using a multiplexed sgRNA expression cassette [97]. Targeting multiple genes in *P. sojae* is still in its early stage as it requires development of multiple Pol III promoter containing a sgRNA cassette. To develop such multiplex cassettes, several factors need to be optimized. First and foremost, multiple RNA Pol III promoters in *P. sojae* need to be identified, and so far, the promoter *RLP41* from *P. sojae* has been successfully used to express single sgRNA [8]. To target multiple genes, more Pol III promoters are necessary. Moreover, newly identified pol III promoters should be smaller in size.

## 7. Hairy Root Technique to Study Gene Function in Soybean

The hairy root technique has been exploited to study the soybean-*P. sojae* interaction [98,99,100,101]. Hairy roots are neoplastic growth that forms following infection with the symbiotic bacterium *Agrobacterium rhizogenes.* Upon infection, *A. rhizogenes* transfers T-DNA from an Ri plasmid into plant cells, which results in the generation of excessive transformed roots. Hairy root technology has been utilized to produce phytochemicals and recombinant proteins, to identify novel genes, and to study host-pathogen interactions [102,103,104]. Hairy root cultures are particularly convenient to study soybean interactions with soilborne pathogens such as *P. sojae*, the soybean cyst nematode, and *Fusarium solani* [105,106]. Recently, Lin et al. (2021) showed that PsAvr1d interacted with GmPUB13 and suppressed self-ubiquitination of GmPUB13, resulting in *Phytophthora* infection [54]. Using soybean hairy roots, overexpression of Ps*Avr1d* promoted *P. sojae* infection and silencing of *GmPUB13* by RNAi decreased infection. GmPUB13 was shown to act as a susceptibility gene with *P. sojae* [54]. Similarly, overexpressing *PsAvh181* of *P. sojae* in soybean hairy roots resulted in disease promotion and silencing of its interacting partner GmSNAPs, which also resulted in disease development [107]. Expression of *PsAvh23* or RNAi silencing of *GmADA2/GmGCN5* in hairy roots enhanced *P. sojae* infection in soybean [34]. Hairy root technology is an excellent tool to elucidate the functional role of effectors and their interacting partners particularly with soilborne pathogens and their host.

Knock out or knock down of a candidate gene followed by phenotyping assays is an essential prerequisite for functional characterization. Currently, there are different phenotyping approaches that are being exploited such as hypocotyl inoculation, root and detached leaf inoculation, and hydroponic assays [108]. *Phytophthora sojae*-soybean research community should come together and agree on unified phenotypic method for effector and host protein functional characterization. Whether effector perception and corresponding downstream signalling pathway in root and leaf need not be conserved. In that case inoculating a root pathogen on leaf to validate the function of a candidate effector may not be an actual outcome.

## 8. Arms Race of Resistance Protein and Effector Interactions

To prevent *P. sojae* infection, genetic resistance has been a key method [5]. However, when novel *Rps* genes are introduced into elite germplasm, the *P. sojae* rapidly evolves by adaptive changes to their *Avr* genes to overcome resistance genes thereby limiting the effectiveness of an *Rps* gene [109,110]. Therefore, there is a constant need for novel *Rps* genes that can effectively limit the disease. Soybean cultivars resistant to distinct *P. sojae* pathotypes, i.e., those bearing specific effector (s) that could be recognised in a gene for gene interaction, have been thoroughly studied [94,111]. So far, more than 30 major *Rps* genes/alleles have been discovered in soybean and about 12 *Avr* genes have been identified and characterized in *P. sojae* (Table 2) [87,112,113,114,115,116,117,118,119,120]. The *Rps* genes likely encode receptors that probably identify *P*. *sojae* effectors and stimulate effector-triggered immunity. To date, all the known oomycete avirulence (*Avr*) genes encode RxLR effectors and the only cloned *Rps* gene, *Rps11*, encodes a coiled-coil-nucleotide-binding leucine-rich repeat (CC-NB-LRR) [52]. It is worth noting that none of the known *Avr* genes code for CRNs. It would be interesting to see if the necrosis phenotype generated by some CRNs is linked to the plant defense response, and if so, whether NLR proteins activate the cell death response.

In commercial soybean cultivars, *Rps* genes have been integrated into breeding efforts to provide resistance against *P. sojae* infection [121]. This all-or-nothing response puts *P. sojae* under selection pressure to overcome *Rps*-mediated resistance. Gain of virulence is frequently associated with mutations that result in the *Avr* gene deletion, amino acids changes, frame shifts mutations and pseudogenization of specific effector genes are also used by *P. sojae* to evade host detection [119,120,122]. Pathogen adaptation has also resulted in polymorphisms in numerous *P. sojae Avr* genes [123,124]. Effector polymorphisms in *P. sojae* also include gene copy number variation and epialleles, in addition to sequence diversification [88,125]. Epigenetic modulation of effector gene expression at the transcription level is thought to make it simpler to recover the virulence capabilities given by *Avr* genes, which is favourable to a pathogen population since it allows effectors to be reused in the absence of *Rps* genes [122].

## 9. Future Perspectives

With the substantial increase in soybean acreage in many countries and particularly in Canada, *P. sojae* has become one of the most devastating pathogens of this crop worldwide [1]. As reviewed here, in just few years, there have been many advances in the plant-microbe interaction research field. Deep learning-based image and sequence analysis will continue to be significant in studying *P. sojae* effectors and those of other pathogens. Since, the proteomic-based subcellular localization information is becoming more popular, the need of computationally predicted localization of unknown proteins is becoming less important. We believe that computational methods should become even more important for elucidating protein structures, the composition of protein complexes and interacting surfaces, dynamics, and interactions between proteins. Despite the report of more than 33 *Rps* genes/alleles in soybean [126], only a few are introgressed in commercial lines (Rps1a, 1c, 1k, 3a and 6) [5]. As a result, the repeated use of the same *Rps* genes has diversified the virulence profile of *P. sojae* through selection pressure [5,127]. How a pathogen adapts to new or existing hosts in response to changing environments is not well studied. With new molecular tools allowing timely and rapid pathogen identification, efficient surveillance strategies will help monitor the evolution of pathogen virulence profiles [124]. Integrating omics (genomics, transcriptomics, proteomics and metabolomics) approaches to identify avr/Avr genes from *P. sojae* and their corresponding Rps genes from soybean will contribute to a better inclusion of resisatnce genes in soybean breeding programs. The recent advancements and reduced costs in sequencing technologies should contribute to a better characterization of effector genes and identification of *Rps* genes. Efficient tools for the functional validation of these newly identified candidate genes are becoming more accessible. For instance, CRISPR-Cas systems have been developed to knock out candidate genes both in *P. sojae* and soybean and should be optimized in the future. One can envision that knock-out of multiple candidates of the same gene family through CRISPR-Cas could be exploited to accelerate functional studies of effector and *Rps* genes. Development of a *P. sojae* effector-R protein interacting network is largely understudied. Protein-protein interacting maps will help in identifying new and novel resistance proteins. Validating the interaction between newly identified *P. sojae* effectors and an R protein in soybean is challenging and time-consuming process and will benefit from faster transformation methods such as the hairy root technique that is amenable to root pathogens. In conclusion, a better understanding of *P. sojae* effectors and their targets in soybean is critical for the development of durable disease resistance.

## Figures and Tables

**Table 1:** Subcellular localization and target proteins of *Phytophthora sojae* effectors assayed in heterologous systems or natural host.

Effector	Cellular Localization	Tag	Expression In	Target	Reference
PsXEG1	Apoplast	PsXEG1-HA-His	*N. benthamiana*	GmGIP1	[48,49]
PsXLP1	Apoplast	PsXLP1- HA-His	*N. benthamiana*	GmGIP1	[49]
PsAvr6	Apoplast	PsAvr6-mCherry	Stable transformation in *P. sojae* followed by soybean root inoculation	Unknown	[32]
PsAvh240	Plasma membrane	GFP-PsAvh240	*N. benthamiana*	GmAp1	[50]
PsAvh241	Plasma membrane	GFP-PsAvh241	*Arabidopsis*, Onion, and *N. benthamiana*	Unknown	[51]
PsAvh181	Plasma membrane	GFP-PsAvh181	*N. benthamiana*	GmSNAP1	[52]
PsCRN78	Plasma membrane	GFP-PsCRN78	*N. benthamiana*	GmPIP2	[47]
PsAvh262	Endoplasmic reticulum	GFP-PsAvh262	*N. benthamiana*	GmBiPs	[53]
PsAvr1d	Cytoplasm	PsAvr1d-mRFP	Stable transformation in *P. sojae* followed by soybean root inoculation	GmPUB13	[54]
PsAvh238	Nucleus and cytoplasm	GFP-PsAvh238	*N. benthamiana*	GmACSs	[44]
PsAvh110	Nucleus	GFP-PsAvh110	*N. benthamiana*	GmLHP1-2	[40]
PsAvh52	Nucleus	PsAvh52-RFP	*N. benthamiana*	GmTAP2	[41]
PsAvr1b	Nucleus	PsAvr1d-YFP	Onion epidermal cells	GmPUB1	[42]
PsAvh23	Nucleus	GFP-PsAvh23	*N. benthamiana*	GmADA2	[34]
PsPSR1	Nucleus	PsPSR1-YFP	*N. benthamiana*	PINP1	[35,36]
PsCRN108	Nucleus	GFP-PsCRN108	*N. benthamiana*	HSE	[38]
PsCRN70	Nucleus	GFP-PsCRN70	*N. benthamiana*	Unknown	[37]
PsCRN63	Nucleus	GFP-PsCRN63	*N. benthamiana*	CATs	[39]
PsCRN115	Nucleus	GFP-PsCRN115	*N. benthamiana*	CATs	[39]
PsAvr3c	Nucleus	GFP-PsAvr3c	*N. benthamiana*	GmSKRP1	[33]
PsFYVE1	Nucleus	YFP-PsFYVE1	*N. benthamiana*	PI3P	[43]

**Table 2:** Reports of interaction between the *Avr* genes of *Phytophthora sojae* and *Rps* genes in soybean.

*Rps* Genes	*Avr* Genes	How the Interaction was Studied	References
*Rps1a*	*Avr1a*	Transient expression of *Avr1a* on *Rps1a* containing soybean plant	[117]
*Rps1b*	*Avr1b*	Positional cloning	[118]
*Rps1k*	*Avr1b-1*	Transient expression of *Avr1b-1* on *Rps1k* and *Rps1b* containing soybean plant	[119]
*Rps1c*	*Avr1c*	Transient expression of *Avr1c* on *Rps1c* plants	[117]
*Rps1d*	*Avr1d*	Transient expression of *Avr1d* on *Rps1d* plants	[120]
*Rps1k*	*Avr1k*	Transient expression of *Avr1k* on *Rps1k* plants	[119]
*Rps3*	*Avr3a*	Transient expression of *Avr3a/5* on *Rps3* plants	[113]
*Rps3a* and *Rps8*	*Avr3a*	CRISPR/Cas9- mediated genome editing of *Avr3a* containing *P. sojae*	[94]
*Rps3b*	*Avr3b*	Transient expression of *Avr3b* on *Rps3b* containing soybean plant	[114]
*Rps3c*	*Avr3c*	Transient expression of *Avr3c* on *Rps3c* containing soybean plant	[112]
*Rps4* and *Rps6*	*Avr4/6*	Transient expression of *Avr4/6* on *Rps4* or *Rps6* containing soybean plant	[86]
*Rps5*	*Avr5*	Transient expression of *Avr3a/5* on *Rps5* containing soybean plant	[86]

## Data Availability

All the data are present in the manuscript.
